# Effect of Plasma pretreatment and Graphene oxide ratios on the transport properties of PVA/PVP membranes for fuel cells

**DOI:** 10.1038/s41598-024-51237-x

**Published:** 2024-01-11

**Authors:** M. O. Abdel-Hamed, Aya A. Draz, Mohamed Khalaf, F. M. El-Hossary, Hamdy F. M. Mohamed, E. E. Abdel-Hady

**Affiliations:** 1 Physics Department, Faculty of Science, Minia University, P.O. Box 61519, Minia, Egypt; 2 Physics Department, Faculty of Science, Sohag University, P.O. Box 82524 Sohag, Egypt

**Keywords:** Materials science, Physics

## Abstract

In this study, a novel proton-conducting polymer electrolyte membrane based on a mixture of polyvinyl alcohol (PVA)/polyvinyl pyrrolidone (PVP) (1:1) mixed with different ratios of graphene oxide (GO) and plasma-treated was successfully synthesized. Dielectric barrier dielectric (DBD) plasma was used to treat the prepared samples at various dose rates (2, 4, 6, 7, 8, and 9 min) and at fixed power input (2 kV, 50 kHz). The treated samples (PVA/PVP:GO wt%) were soaked in a solution of styrene and tetrahydrofuran (70:30 wt%) with 5 × 10^−3^ g of benzoyl peroxide as an initiator in an oven at 60 °C for 12 h and then sulfonated to create protonic membranes (PVA/PVP-g-PSSA:GO). The impacts of graphene oxide (GO) on the physical, chemical, and electrochemical properties of plasma-treated PVA/PVP-g-PSSA:x wt% GO membranes (x = 0, 0.1, 0.2, and 0.3) were investigated using different techniques. SEM results showed a better dispersion of nanocomposite-prepared membranes; whereas the AFM results showed an increase in total roughness with increasing the content of GO. FTIR spectra provide more information about the structural variation arising from the grafting and sulfonation processes to confirm their occurrence. The X-ray diffraction pattern showed that the PVA/PVP-g-PSSA:x wt% GO composite is semi-crystalline. As the level of GO mixing rises, the crystallinity of the mixes decreases. According to the TGA curve, the PVA/PVP-g-PSSA:x wt% GO membranes are chemically stable up to 180 °C which is suitable for proton exchange membrane fuel cells. Water uptake (WU) was also measured and found to decrease from 87.6 to 63.3% at equilibrium with increasing GO content. Ion exchange capacity (IEC) was calculated, and the maximum IEC value was 1.91 meq/g for the PVA/PVP-g-PSSA: 0.3 wt% GO composite membrane. At room temperature, the maximum proton conductivity was 98.9 mS/cm for PVA/PVP-g-PSSA: 0.3 wt% GO membrane. In addition, the same sample recorded a methanol permeability of 1.03 × 10^−7^ cm^2^/s, which is much less than that of Nafion NR-212 (1.63 × 10^−6^ cm^2^/s). These results imply potential applications for modified polyelectrolytic membranes in fuel cell technology.

## Introduction

The fuel cell (FC) is an important new energy source. It is an electrochemical energy source that transfers the chemical energy inherent in chemical bonds into electrical energy via a redox reaction. Fuel cells are expected to be one of the next-generation power source technologies for future portable and fixed stationary applications. They have been one of the forerunners in the field of alternative energy in recent years since they are a clean source of energy with great efficiency and power density^1–3^.

Both polymer electrolyte membrane fuel cells (PEMFCs) and direct methanol fuel cells (DMFCs) use a polymer electrolyte membrane (PEM) that is connected to electrodes as a membrane electrode assembly (MEA) to act as a proton conductor. The PEM is the fuel cell's core, carrying protons from the anode to the cathode and letting the electrons pass through an external circuit^4^. DMFCs and PEMFCs are gaining popularity as environmentally friendly energy sources with high power density, which is essential for use in electric vehicles and residential power generators^5–11^. However, numerous issues must be addressed before large-scale commercialization can take place, including the high cost and thermal stability of the PEM. PEM characteristics are thought to have changed as a result of contamination or the formation of crosslinked sulfonic anhydrides through condensation of side-chain sulfonic acid (SO_3_^−^) groups^12–17^. Three technical drawbacks of Nafion NR-212 prevent it from being widely used in fuel cell applications, despite its good chemical and physical properties: high cost (manufactured through an expensive and complex process), low conductivity at high temperatures or low humidity, and high methanol permeability, which lowers cell efficiency^18^. As a result, their replacement with cost-effective polymer membranes is extremely desirable.

Polymer mixing is a green chemistry technique for producing innovative polymeric materials with all of the features of pure polymers^19^ to improve the electrical, mechanical, and electrochemical properties of polymer electrolyte membranes. Polyvinyl alcohol (PVA) is a low-cost, widely used polymer with good chemical stability and hydrophilicity that enables easy film formation but has lower ionic conductivity. To overcome this disadvantage, mixing PVA with other polymers or adding dopants is required. A polymer that is usually mixed with PVA is polyvinyl pyrrolidone (PVP) because hydrogen bonds are formed between the hydroxyl side groups of PVA and the carbonyl groups in PVP^20^. PVP is environmentally friendly and has an amorphous property compared to other semi-crystalline polymers, which is advantageous when blended with PVA and leads to a reduction in the crystallinity of PVA, thereby improving the ionic conductivity of PVA/PVP membranes. In addition, PVA/PVP blend membranes appear to have higher oxidation stability but are more prone to mechanical failures because they can absorb more water^21^. It was reported by Zidan et al. that the crystallinity of PVA/PVP blends decreases with increasing the level of PVP and PVA (50) wt%/PVP (50) wt% is more stable and suitable samples for investigation due to its brittleness^22^.

The exciting aspect of combining PVA and PVP polymers is that, by incorporating different nanoparticles, their properties can be modified to create unique nanocomposites (NCs). PVA/PVP mix NCs with varying ratios of CuO nanoparticles as suitable filler were constructed by Mallakpour et al.^23^ to create the requisite blend NCs to accomplish the required properties and analyze antibacterial activity. They discovered that the manufactured NC membranes have excellent thermal and optical properties and might be applied to medical operations to cure bacterial infections. In a different study, Rajesh et al.^24^ reported the production of PVA/PVP nanocomposites with different concentrations of titanium dioxide by solvent casting. Their outcomes showed the improvement of PVA/PVP-TiO_2_ nanocomposite memory's mechanical, electrical, optical, and dielectric properties. PVA/PVP blend-loaded magnesia nanoribbons were fabricated by El-Gamal et al.^25^, utilizing various ratios to regulate the characteristics of the nanocomposite materials and discover fresh uses for the generated nanocomposite membranes. Films of PVA-PVP blended and doped with cadmium chloride (CdCl_2_) from 0 to 40 wt% were created by Baraker et al.^26^. They found that when CdCl_2_ is added as the dopant, samples of the PVA-PVP polymeric mix exhibit a substantial fluctuation in DC electrical conductivity.

Since its discovery, Graphene, a monolayer of hexagonally packed carbon atoms, has revolutionized both the academic and industrial worlds^27^. Graphene/polymer composites have sparked a lot of interest due to their wide range of applications in high-strength and conductive materials, catalysts, and energy-related systems, particularly flexible energy conversion and storage devices. Furthermore, the low cost of manufacture (graphite) and the exceptional characteristics of graphene sparked significant fascination with producing polymer composite materials that are low-cost and high-performance^28^. The existence of hydrophilic functional groups in GO generated an easy environment for proton conduction via a "hopping" process, which boosted water retention, which is required for proton conduction in non-humid environments. Because of its enhanced proton conductivity and water retention capability, GO has been recommended as an appealing organic filler for (PEM)^29,30^. A nanohybrid anhydrous PEM based on phosphorylated GO (PGO)/chitosan (CS) was stated for increased-temperature FCs^31^. The mechanical properties and thermal stability of a CS membrane were expressively enhanced by a PGO-doped nanohybrid PEM. At 160 °C, the CS/PGO-2.5 (2.5% PGO) nanohybrid PEM demonstrated 5.79 mS/cm proton conductivity and 0% relative humidity (RH) retention ability. Sharma and Kulshrestha^32^ developed a highly stable and water-retentive sulfonated CS (SCS) modified with a GO nanohybrid membrane, which led to enhanced thermal and mechanical stabilities as well as a proton conductivity of 112 mS/cm at 90 °C. A composite membrane was created and tested using sulfonated graphene oxide (s-GO) and sulfonated polyether ether ketone (SPEEK) with varying sulfonated graphene oxide concentrations by Heo et al.^33^. The researchers discovered that adding s-GO particles to the SPEEK membrane enhanced its proton conductivity by up to 8.41 mS/cm. Additionally, it was demonstrated that the s-GO/SPEEK membranes had better selectivity than Nafion NR-212 and reduced methanol permeability (2.63 × 10^−7^ cm^2^/s). Neelakandan et al.^34^ created PEMs using sulfonated poly (1,4-phenylene ether ether sulfone) (SPEES)/poly(ether imide) (PEI)/sulfonated graphene oxide (s-GO). They discovered that as the s-GO level grew, the composite membranes' tensile strength and proton conductivity increased. The addition of 0.8 wt% s-GO resulted in a maximum conductivity of 8.87 mS/cm at 25 °C. The methanol permeability of all SP/s-GO membranes was less than 3.26 × 10^−7^ cm^2^/s.

The aim of this work is to employ the different percentages of PVA/PVP-g-PSSA:x wt% GO (x = 0.1, 0.2, and 0.3) nanocomposite membranes to achieve high proton conductivity and low methanol permeability. Additionally, it evaluates how well the composite membranes function in terms of water uptake, swelling ratio, and IEC. To the best of our knowledge, this system based on grafted PVA/PVP employing the plasma technique has never been the subject of prior research. Herein, the Dielectric Barrier Dielectric (DBD) plasma treatment was used to investigate the effect of plasma processing duration on the characteristics of PVA/PVP-x wt% GO. Plasma processing time was limited to 0–9 min at a fixed plasma power of 2 kV in order to optimize the treatment process and thus acquire optimal membrane characteristics. SEM, XRD, and FTIR have all been used to characterize the prepared polymer electrolytes in order to examine their morphology, structure, vibration, and electrical characteristics.

## Experimental and methods

### Materials and chemicals

Hayashi Pure Chemical Industries Ltd., Japan, provided polyvinyl alcohol (PVA) (CH_2_CH(OH))_n_ with a molecular weight of 1700, while Techno Pharmachem Haryana, India, provided polyvinyl pyrrolidone (PVP) (C_6_H_9_NO)n with a molecular weight of roughly 36,000. Styrene solution (99.9%) was purchased from Alfa Aesar. Graphite powder (99.5%) was supplied by Central Drug House (P) Ltd., and concentrated sulfuric acid (H_2_SO_4_) with Mw (98.07) was procured from Elnasr Pharmaceutical Chemicals Co. Potassium persulphate K_2_S_2_O_8_ (98%), phosphorus pentoxide P_2_O_5_ (98%), and Potassium permanganate KMnO_4_ (99%) were supplied by Pratap Chemical Industries Pvt. Ltd.

### Membranes fabrication

#### Preparation of graphene oxide (GO): (Hummer-Offemen) method

**Figure 1:**
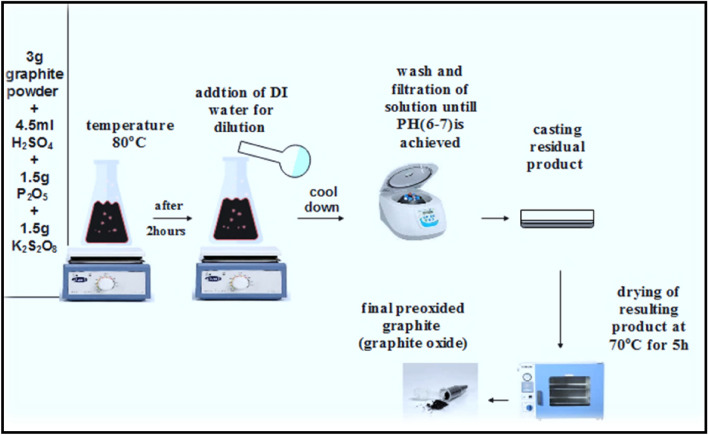
Schematic of preparation method for peroxided GO.

**Figure 2:**
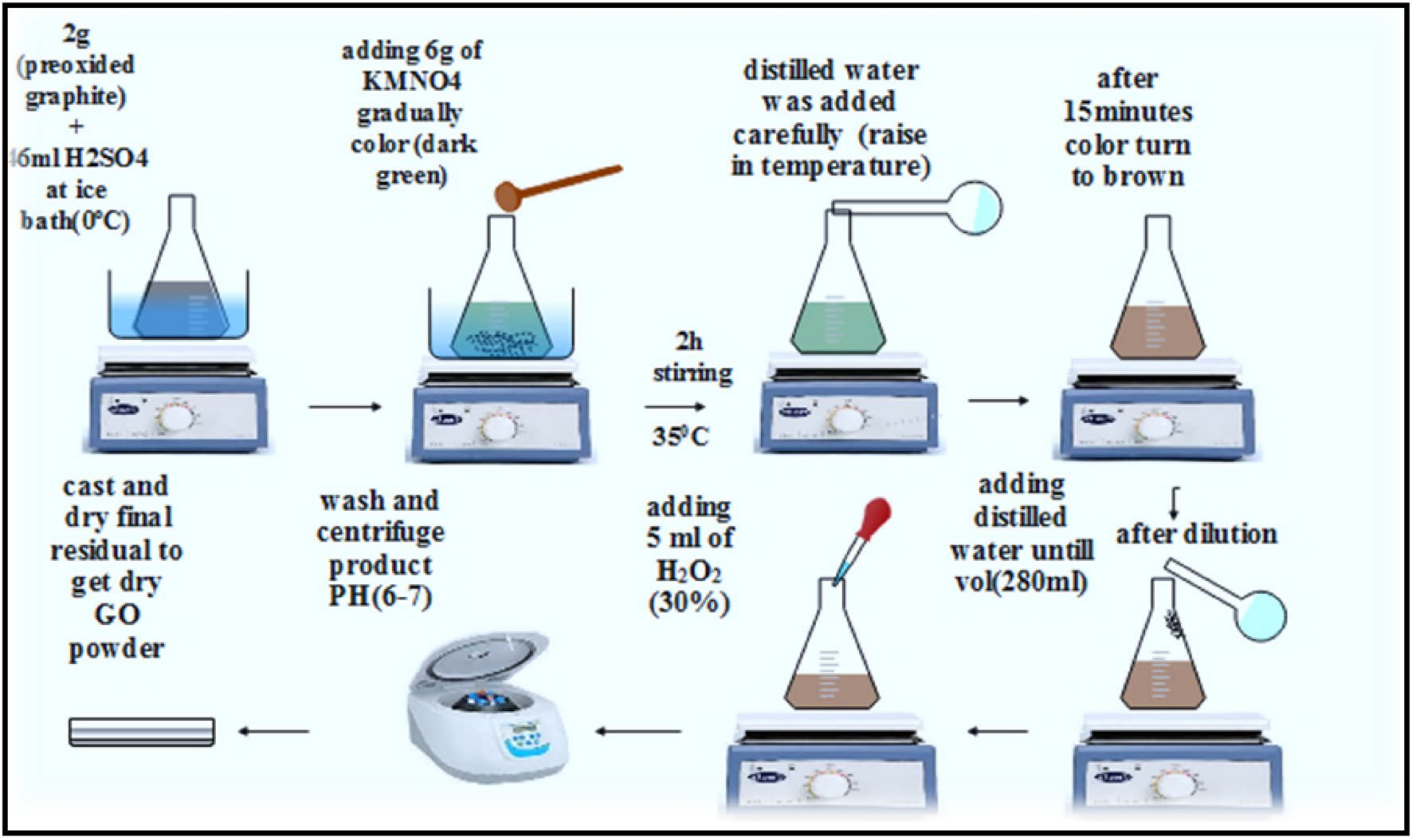
Schematic diagram of graphene oxide (GO) preparation method.

As can be seen in Fig. 1, 4.5 ml of concentrated H_2_SO_4_ was mixed with 3 g of pure graphite, 1.5 g of potassium persulfate K_2_S_2_O_8_, and 1.5 g of phosphorus pentoxide P_2_O_5_ at 80 °C. The mixture was stirred for two hours, and then let it cool. After centrifuging, diluting, and carefully washing the resulting solution with deionized water, the solution's pH was adjusted to 6–7. As illustrated in Fig. 2, while keeping the temperature below 20 °C, 2 g of graphite peroxide was added to 46 ml of concentrated H_2_SO_4_ acid and 6 g of potassium permanganate (KMnO_4_) in an ice bath (0 °C) and progressively stirred. When warm distilled water was added, strong foaming occurred after the mixture was heated to 35 °C and thoroughly stirred for two hours (dark green color), and the mixture's temperature increased to 98 °C. The mixture was then continuously stirred for 15 min. After that, 280 ml of warm distilled water was added to the mixture to dilute it. The diluted solution was then combined with 5 ml of 30% H_2_O_2_. The final step involved centrifuging the prepared mixture until the pH was verified for neutrality, and then the resulting solution was dried at 60 °C in an oven.

#### Preparation of PVA/PVP: GO nanocomposite membranes

Aqueous PVA solution was prepared by dissolving 0.6 g PVA in deionized water and then heating at 70 °C for 48 h. The PVP (0.6 g) was mixed with the PVA solution, and the mixture was vigorously stirred at room temperature for 24 h. Graphene oxide nanoparticles were slowly added to the homogenous polymer mixture in weight percentages of 0.0, 0.1, 0.2, and 0.3, and then the mixture was left to stir for an additional period of 12 h. Finally, each solution was sonicated for 20 min to obtain a good dispersion of graphene oxide in the blended polymer. The solution was then poured onto a petri dish and placed in a hot air oven set to 70 °C. Fine nanocomposite polymer membranes with the combinations (PVA/PVP:xGO) (x = 0.0, 0.1, 0.2, and 0.3 wt%) were obtained, which were kept in a desiccator until further testing.

### Grafting and sulfonation of PVA/PVP:xGO composite membranes

#### Styrene grafting on membranes

**Figure 3:**
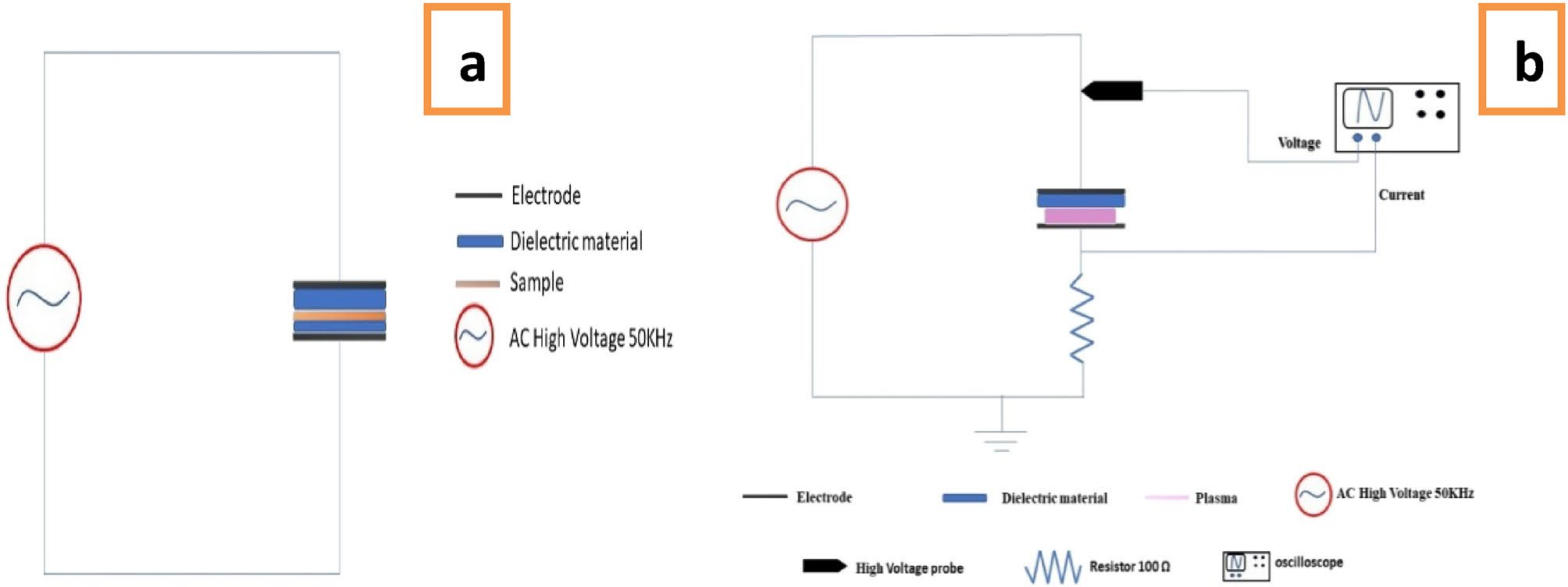
(**a**) Schematic diagram of DBD device, and (**b**) Current–voltage circuit diagram of atmospheric pressure plasma.

Dielectric barrier dielectric (DBD) plasma was used to activate the surface, form surface functional groups, and increase the surface energy of the sample. Plasma first treated the surface of the membrane by producing free radicals on the surface to smooth the grafting process. A variable high-voltage (2 kV, 30 mA) power supply with a frequency of 50 kHz was used to create dielectric barrier dielectric (DBD) plasma. The DBD plasma reactor consists of two stainless-steel electrodes with an area of 2 cm^2^ and a thickness of 1 mm. One was covered with a 1 mm thick polymer plate, and the other was covered with a 0.1 mm thick polymer plate, as shown in Fig. 3a. A two-channel digital oscilloscope was used to examine the high voltage and current waveforms of the DBD plasma system. For voltage recording, a high-voltage probe (1000:1) was used, and it was connected in parallel to the plasma electrode. To detect the discharge current, A 100 Ω resistance was placed between the ground terminal and the ground electrode and assigned to the other channel. By substituting the resistance with 10nF capacitor, the Lissajous relationship can be used to calculate dissipated plasma power measurements from the Q–V relationship (Fig. 3b). Plasma was used to process the prepared samples at various dose rates (2, 4, 6, 7, 8, and 9 min), after which the treated samples were grafted and sulfonated to create protonic membranes.

In this work, the prepared membrane was grafted using a two-step procedure. Firstly, plasma treatment was performed with different time doses of plasma (2, 4, 6, 7, 8, and 9 min), and then the membranes were weighed. Secondly, those membranes were soaked in a solution of styrene and tetrahydrofuran (70:30 wt%) with 5 × 10^−3^ g of benzoyl peroxide as an initiator in an oven at 60 °C for 12 h. Styrene-grafted nanocomposite polymer membranes with combinations of PVA/PVP:x wt% GO were obtained.

#### Sulfonation of grafted PVA/PVP: GO nanocomposite membranes

Concentrated sulfuric acid H_2_SO_4_ was diluted to 0.02 M by mixing it with 20 M of tetrahydrofuran during the sulfonation process. The grafted membrane was left for 2 h at 40 °C in a sulfuric acid solution, and the solution then remained at ambient temperature for 24 h. After that, the sulfonated membrane sample was washed with tetrahydrofuran to remove any impurities. The resulting samples were weighed several times until an approximate fixed weight was obtained. Finally, after sulfonation, proton-conducting polymer electrolyte membranes with combinations of PVA/PVP-g-PSSA:x wt% GO were obtained. Nafion NR-212 was used for comparison with PVA/PVP-g-PSSA:x wt% GO. All Nafion NR-212 membranes were treated in accordance with the following steps: 1 h in a 3% hydrogen peroxide (H_2_O_2_) solution at 80 °C, followed by an hour in deionized water at 80 °C to remove any remaining H_2_O_2_ traces, followed by an hour in 0.02 M H_2_SO_4_ at 80 °C, and finally an hour in deionized water at 80 °C. After being treated, the membranes were then immersed in deionized water until they were used^35^.

## Characterization

The surface free energy was investigated by calculating the wetting parameter from Young’s equation

1 $$\upomega =\frac{{\upgamma }_{{\text{GO}}/{\text{PVA}}}-{\upgamma }_{{\text{GO}}/{\text{PVP}}}}{{\upgamma }_{{\text{PVA}}/{\text{PVP}}}}.$$

where γ_GO/PVA_ is the interfacial tension between GO and PVA, γ_GO/PVP_ is the interfacial tension between GO and PVP, and γ_PVA/PVP_ is the interfacial tension between PVA and PVP. The interfacial tension γ_12_ between different components can be determined as the Harmonic-mean equation.

2 $${\gamma }_{12}={\gamma }_{1}+{\gamma }_{2}-4\frac{{\gamma }_{1}^{d}{\gamma }_{2}^{d}}{{\gamma }_{1}^{d}+{\gamma }_{2}^{d}}+\frac{{\gamma }_{1}^{p}{\gamma }_{2}^{p}}{{\gamma }_{1}^{p}+{\gamma }_{2}^{p}}.$$

Geometric-mean equation

3 $${\upgamma }_{12}={\upgamma }_{1}+{\upgamma }_{2}-2\left[\sqrt{{\upgamma }_{1}^{{\text{d}}}{\upgamma }_{2}^{{\text{d}}}}+\sqrt{{\upgamma }_{1}^{{\text{p}}}{\upgamma }_{2}^{{\text{p}}}}\right].$$

where γ_1_ and γ_2_ are the free surface energy of mixture components 1 and 2, respectively, ${\gamma }_{1}^{d}$ and ${\gamma }_{2}^{d}$ are the dispersive parts of the free surface energy of the mixture components, respectively, and ${\gamma }_{1}^{p}$ and ${\gamma }_{2}^{p}$ are the polar parts of the surface free energy of the mixture components, respectively.

After plasma treatment and grafting processes, the membrane samples were reweighed to give a degree of grafting (DOG), which was calculated from the following equation^36^.

4 $${\text{DOG}}\left(\%\right)=\frac{{W}_{g}-{W}_{s}}{{W}_{s}} \times 100$$

where *W*_*g*_ is the weight of the sample after grafting, *W*_*s*_ is the weight of the sample before grafting.

The sulfonated samples were weighed several times until an approximate fixed weight was obtained. The degree of sulfonation (DOS) was calculated from equation^37^.

5 $${\text{DOS}}\left(\%\right)=\frac{{W}_{wet}-{W}_{dry}}{{W}_{dry}}\times 100.$$

where *W*_*wet*_ is sulfonated sample weight and *W*_*dry*_ is the dry sample weight.

XRD was done on PANalytical (Empyrean) by using CuKα radiation (wavelength of 0.154 nm) at an accelerating voltage of 40 kV and a current of 35 mA. The crystallite size (*D*) of GO was calculated using Deby-Sherrer’s, which is expressed by the following formula:

6 $$D=\frac{0.9\uplambda }{\beta Cos(\theta )}.$$

here β is full width at half maximum (FWHM) at the diffraction angle 2θ, λ = X-ray wavelength. The interatomic spacing was investigated from Bragg’s law as^38–40^

7 $$2dsin\left(\theta \right)= n\lambda .$$

The degree of crystallinity *Xc* for all prepared samples was estimated from Eq. (8)^41^ to determine the impact of GO concentration on the crystalline structure alterations of the host matrix.

8 $${X}_{C}=\frac{{A}_{c}}{{A}_{c}+{A}_{a}}.$$

*A*_*c*_ and *A*_*a*_ were determined from the convolution of the XRD peaks using Fityk software^42^.

To determine the molecular vibration in chemical bonds, FTIR spectra were obtained using a Bruker vertex 70 (series number 1341), and its spectrum was studied in the wavenumber range of 4000–400 cm^−1^. The JSM-IT200 was used to acquire SEM images that could be used to examine the morphologies of the membrane's surface and their stoichiometry. The measurements were performed at the Central Laboratory for Microanalysis and Nanotechnology, Minia University, Egypt.

The thermal characteristics of the membranes were assessed using a thermogravimetric analyzer (TGA) instrument model Q50USA. The sample was heated at a rate of 10 °C per min to 700 °C in a nitrogen environment in order to perform the measurements.

To prepare the dried membrane for water uptake (WU) measurements, membranes with a dimension 1.5 cm^2^ were evacuated for 24 h. The dry membrane was weighed and then immersed in distilled water for 24 h at 25 °C to create a saturated membrane with water. The wet membrane was weighed repeatedly until its weight did not change. The water uptake was then calculated using the following equation^43^:

9 $${\text{Water}} {\text{uptake}}\left(\%\right)=\frac{{W}_{w}-{W}_{d}}{{W}_{d}}\times 100.$$

where *W*_w_ is the weight of the wet (hydrated) membrane and *W*_d_ is the weight of the dry membrane. The difference between the wet and dry dimensions in the length or thickness direction was used to calculate the swelling (*S*) of the membranes, which can be calculated by^43^

10 $$S\left(\%\right)=\frac{{L}_{w}-{L}_{d}}{{L}_{d}}\times 100.$$

where *L*_w_ denotes the length of wet membranes, while *L*_d_ denotes the length of dry membranes, respectively.

The ion exchange capacity (IEC meq/g) of the membranes was determined. Each membrane was cut into square sections with dimensions (2 × 2 cm^2^), then immersed in 20 ml of a 2 M NaCl solution for at least 24 h to substitute sodium ions for protons. Using phenolphthalein as an indicator, a 0.01 M NaOH solution was used to titrate the remaining solution. The IEC test was investigated using the titration method and was computed using the following equation^43^:

11 $$IEC\left(meq/g\right)=\frac{{C}_{NaoH}\times {V}_{NaoH}\times n}{{W}_{d}}.$$

where *C*_*NaOH*_ denotes the concentration of NaOH solution, *V*_*NaOH*_ denotes the volume of NaOH used for neutralization, and *n* denotes the ratio of the amount of NaCl in which the sample was immersed to the total amount used for titration.

The AC proton conductivity σ_ac_ for grafted PVA/PVP, PVA/PVP-g-PSSA:0.1 wt% GO, PVA/PVP-g-PSSA:0.2 wt% GO, and PVA/PVP-g-PSSA:0.3 wt% GO membranes was determined. An LCR meter (Hioki 3532) was used to measure the conductance *G*. The resistance of the prepared membranes was derived from impedance spectra and then transformed into proton conductivity using the following equation^44^.

12 $${\sigma }_{ac}=\frac{GL}{A}.$$

where ${\sigma }_{ac}$ is the conductivity, the thickness of the membrane is denoted by *L*, while the effective area of the blocking electrode is denoted by *A*. The membranes were kept in water for 24 h to be hydrated before the measurements. All of the tests were done at room temperature, and the frequencies ranged from 50 Hz to 5 MHz.

A glass cell was used to measure the methanol permeability that served as a diffusion cell and had two compartments that were the same. Between the two compartments, the composite membranes were placed, and they were firmly clamped. A suitable volume of 3M methanol solution and deionized water were put into compartments A and B, respectively. Then, for 30 min, both compartments were left to stir at room temperature. Every 24 h, 1 mL of compartment B was sampled. The refractive index of the solution in chamber B was determined using the refractometer (2WAJ ABBE), and the methanol concentration was determined. The permeability P of methanol was then calculated using the equation^45^.

13 $${\text{P}}=\frac{mL{V}_{B}}{S{C}_{A}}.$$

where *m* is the slope of the line, it represents the relation between penetration time and methanol concentration *C*_*B*_. The letters *V*_*B*_ and *C*_*A*_ represent the volume of solution in chamber B and the concentration of methanol in chamber A, respectively. The membrane's thickness and compelling surface area are represented by the letters *L* and *S*, respectively.

The following equation, which quantifies proton conductivity and methanol permeability, was used to assess the performance of polyelectrolyte membranes for use in DMFCs^33^

14 $$E=\frac{\sigma }{P}.$$

where *E* represents membrane efficiency, *P* is methanol permeability of the manufactured membranes, and $\sigma$ indicates conductivity.

## Results and discussion

### Surface free energy calculations

In general, the combined influence of thermodynamic and kinetic factors dictates the localization of GO in PVA/PVP blends, as predicted by Young's equation^46^. Based on the surface parameter data in Table 1, GO has a tendency to be localized in PVA when (ω <  − 1), GO lean to be localized in PVP if (ω > 1), and when ω (− 1 < ω < 1), GO is localized between PVA and PVP^47–49^. Using Eq. (2), the ${\gamma }_{Go/PVA}$,${\gamma }_{Go/PVP,}$, and ${\gamma }_{PVA/PVP}$ were 43.4, 20.18, and 7.36 mJ/m^2^, while they were 25.9, 10.67, and 4.01 mJ/m^2^ using Eq. (3). According to Eq. (1), $\omega$ was 3.15 using the harmonic-mean equation and was 4.51 using the geometric-mean equation. So, in this case, the value of $\omega >1$ confirmed that GO tends to be selectively sited in the PVP phase during the melt blending process.

**Table 1:** The value of polar and dispersion parts of PVA, PVP, and GO.

Sample	${{\varvec{\gamma}}}^{{\varvec{s}}}$ (mJ/m^2^)	${{\varvec{\gamma}}}^{{\varvec{d}}}$ (mJ/m^2^)	${{\varvec{\gamma}}}^{{\varvec{p}}}$ (mJ/m^2^)	References
PVA	39.1	34.6	4.4	^47^
PVP	44.7	28.4	16.2	^48^
GO	46.9	38.7	8.3	^49^

### Degree of grafting (DOG)

**Figure 4:**
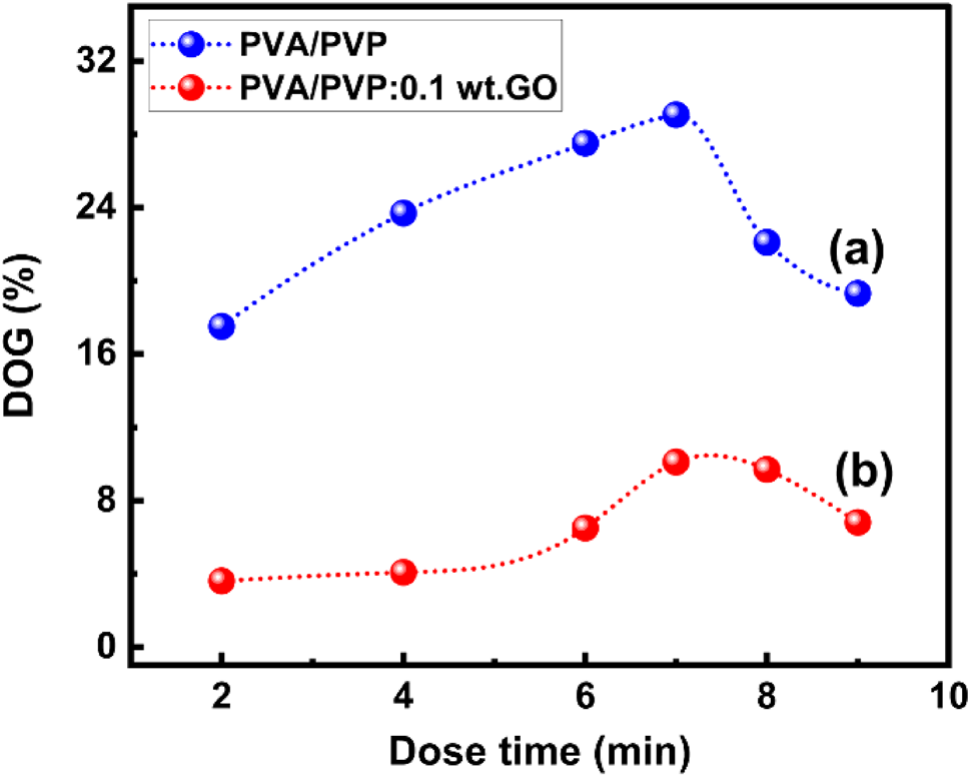
Degree of grafting with time of dose for PVA/PVP pure and PVA/PVP: 0.1 wt% GO membranes.

In the present study, the styrene was grafted onto plasma-treated PVA/PVP: xGO using the solution grafting technique, and the DOG was calculated using Eq. (4). Figure 4a,b shows the effect of the duration of plasma exposure on DOG of styrene on PVA/PVP pure and grafted PVA/PVP: 0.1 wt% GO membranes. This figure shows that the optimal DOG value for both membranes was at the plasma exposure time of 7 min and then decreased, which may be due to the recombination of existing bonds rather than creating free radicals on the surface. The degree of grafting at the 7 min dose was about 29.06%, which is the maximum value of the pure membrane. For grafted PVA/PVP: 0.1 wt% GO membrane, maximum DOG similarly occurred at a plasma dose of 7 min. This is evident in Fig. 4b, where the maximum DOG was 10.7%. This decrease in DOG relative to the pure membrane can be attributed to the reaction of graphene oxide with the side chain bonds of the PVA/PVP membrane, which reduced the chance of generating free radicals on the membrane surface.

### Degree of sulfonation (DOS): effect of time

**Figure 5:**
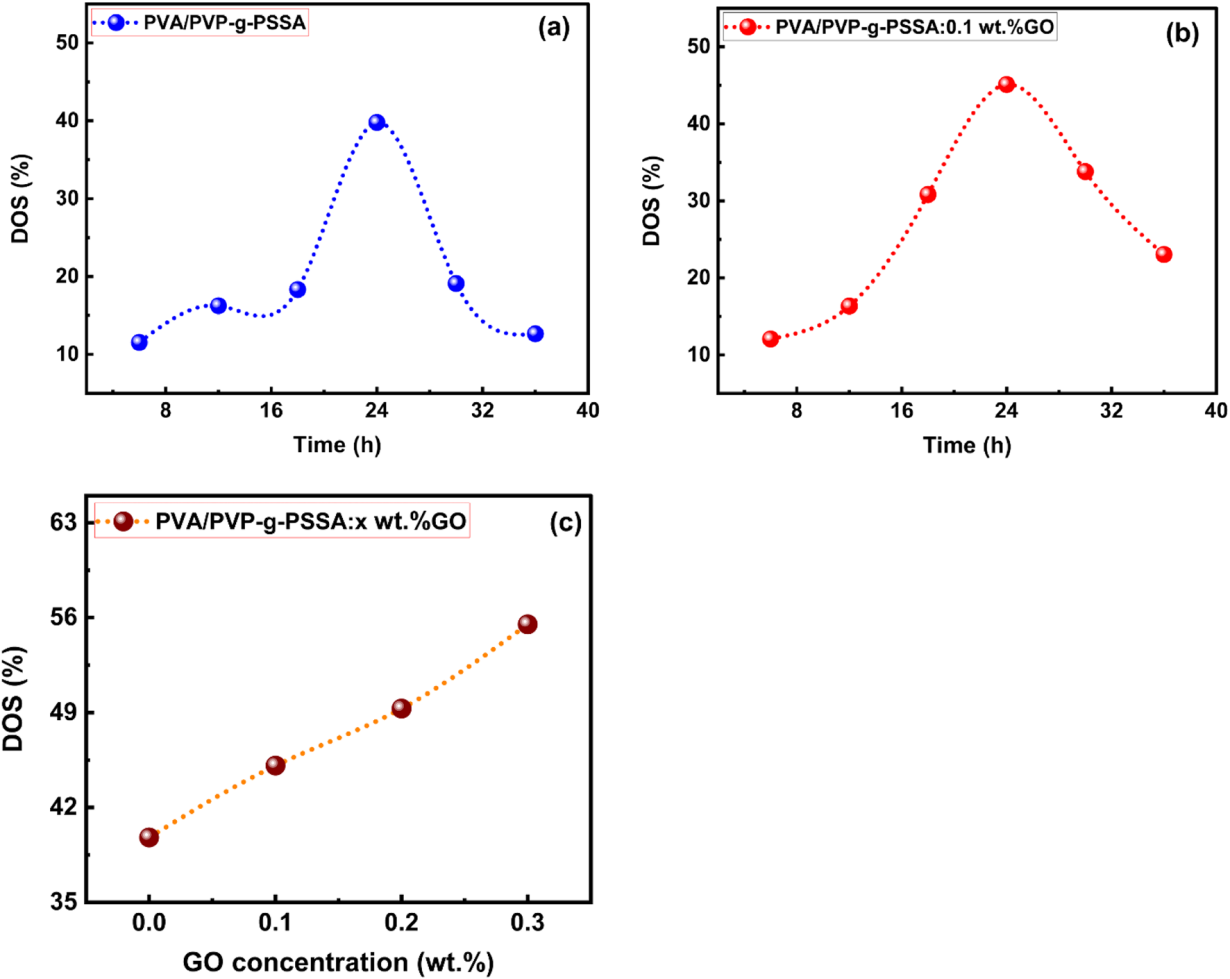
The variation of degree of sulfonation with time (**a**) for the grafted PVA/PVP-g-PSSA pure sample, (**b**) the grafted PVA/PVP-g-PSSA: 0.1 wt% GO composite membrane sample, and (**c**) with concentration of GO.

Ion exchange sites were introduced in the styrene-grafted membranes to be employed as PEMs for power devices by post sulfonation of grafted PVA/PVP (PVA/PVP-g-PSSA) membranes, where sulfonic acid groups (SO_3_) were linked to the aromatic rings of the grafted polystyrene. The sulfonation process was prepared for each of the membrane samples with different time periods (6, 12, 18, 24, 30, and 36 h) in 0.02 M of sulfonic acid, and then the DOS value was obtained using Eq. (5). The maximum DOS was obtained at 24 h for all prepared membranes, as shown in Fig. 5a,b. Figure 5c indicates the variation of DOS with GO concentration, and it was observed that DOS increases as the GO ratio becomes higher.

### FTIR analysis of PVA/PVP-PSSA:x wt% GO composite membrane

**Figure 6:**
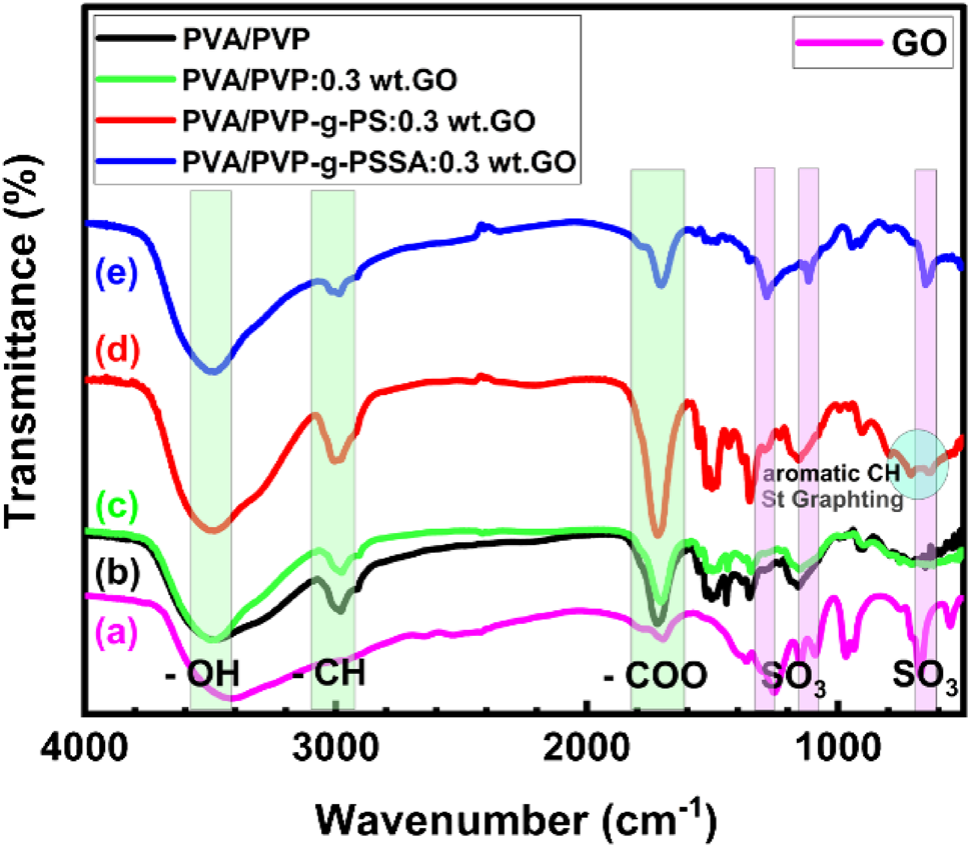
FTIR spectrum of (**a**) GO, (**b**) PVA/PVP pure, (**c**) PVA/PVP doped with 0.3 wt% GO composite, (**d**) styrene grafted PVA/PVP: 0.3 wt% GO and (**e**) sulfonated PVA/PVP-g-PSSA: 0.3 wt% GO membrane.

FTIR analysis is the most effective method to evaluate the composite membrane’s structural and chemical characteristics. In addition, FTIR spectra were employed to confirm the grafting and sulfonation processes of the plasma-pretreated for 7 min PVA/PVP-g-PSSA:x wt% GO composite membranes. Figure 6a–e shows typical FTIR spectra of GO, PVA/PVP pure, PVA/PVP:0.3 wt% GO, PVA/PVP-g-PS:0.3 wt% GO grafted and PVA/PVP-g-PSSA:0.3 wt% GO sulfonated membranes, respectively and Table 2 lists the FTIR vibration bands position. For GO (Fig. 6-a), various oxygen configurations in the structure include the vibration modes of O–H group stretching vibration band at 3395 cm^−1^, the sp2 structure of C=C is observed at 1635 cm^−1^ where the peak at 1177 cm^−1^ is related to the C–O stretching vibration band. The structure of C-H was observed at 869 cm^−1^ and 590 cm^−1^, respectively^50^. Figure 6b–e depicts the PVA/PVP polymeric blend membrane. There is a distinct broadband at 3496 cm^−1^ that corresponds to the PVA/PVP polymeric blend's pure hydroxyl group's O–H stretching vibration^51^. A second band can be found at 2979 cm^−1^ which corresponds to C–H asymmetric stretching^52^ while the appearance of the C=O starching band was at 1725 cm^−1^^53^. The band that appears at 1498 cm^−1^ is observed to be corresponding to C–H bending vibration^54^. For the band observed at 1338 cm^−1^ is owed to (CH + OH)^55^, the C–O–C stretching band is seen at 1163 cm^−1^, whereas out of plane C–H bending band is observed at 908 cm^−1^^56^. Figure 6d shows successful polystyrene grafting on PVA/PVP: the aromatic CH deformation of the mono-substituted benzene ring of grafted polystyrene was given a couple of bands at 740 and 668 cm^−1^ in a 0.3 wt% GO composite membrane^57^. Polystyrene is a mixed molecule, meaning it contains both saturated and unsaturated carbons; in this example, the unsaturated carbons are from the benzene ring. This is indicated by the fact that it has C–H stretches above and below 3000 cm^−1^^5^. Finally in Fig. 6e, the appearance of distinct bands at 1280, 1112, and 638 cm^−1^, respectively^58^, at the grafted film’s interface confirms the sulfonation of benzene rings.

**Table 2:** FTIR vibration bands position.

Wavenumber (cm^−1^)	Vibration type	Assignment
3496	Stretching	O–H
2979	Asymmetric stretching	C–H
1725	Stretching	C=O
1498	bending	C–H
1138	Stretching	C–O–C
1280-1112-638	–	SO_3_

### Morphology of GO and PVA/PVP:x wt% GO polymeric nanocomposite membranes

**Figure 7:**
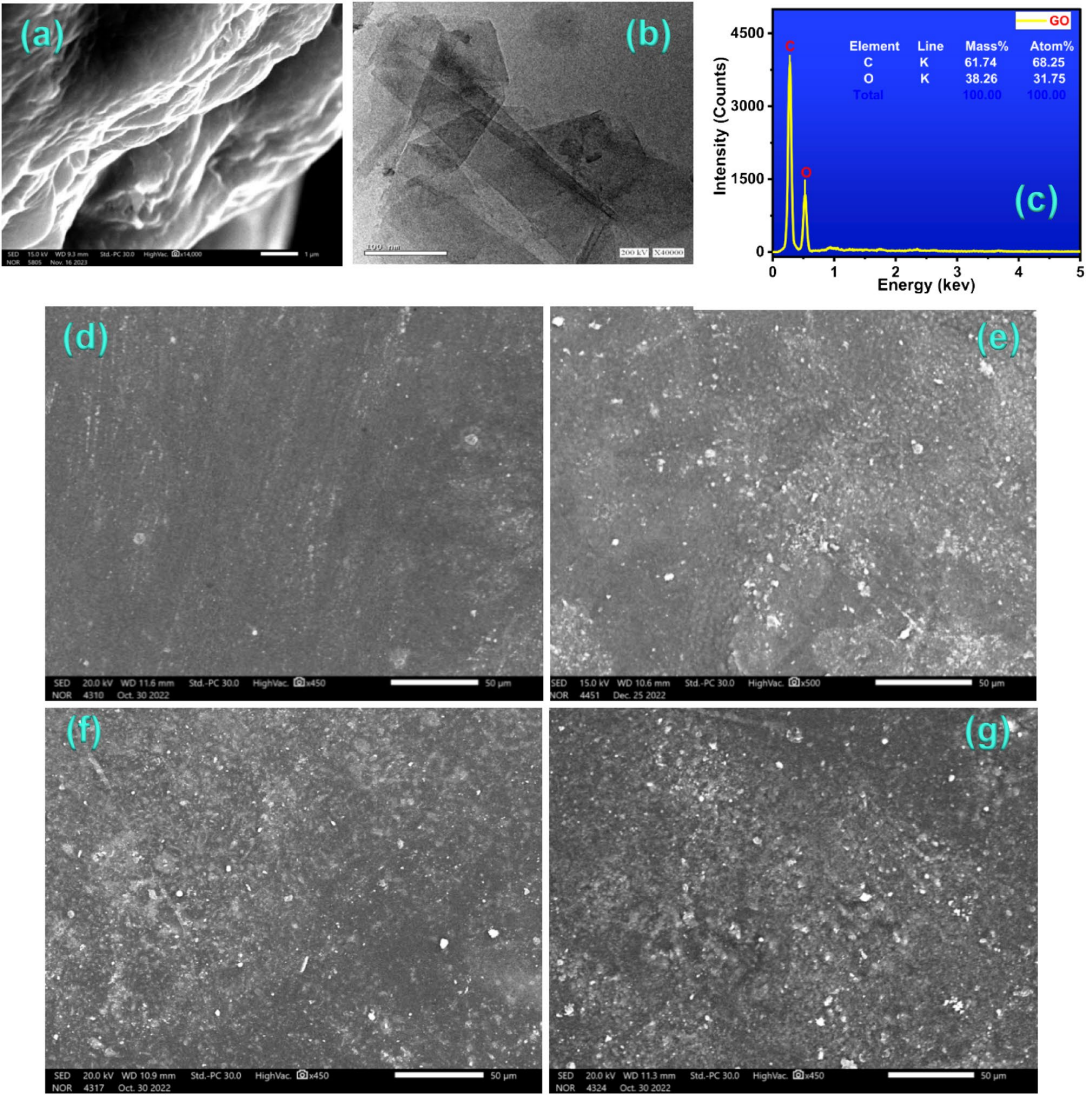
SEM and TEM micrographs of the surface morphology of GO (**a**, **b**) and EDX for GO (**c**) SEM images of the surface morphology of PVA/PVP blended polymers: (**d**) PVA/PVP; (**e**) PVA/PVP: 0.1 wt% GO; (**f**) PVA/PVP: 0.2 wt% GO; and (**g**) PVA/PVP: 0.3 wt% GO composite.

To provide further rigidity and strength, graphite sheets were converted into the corrugated form of GO (Fig. 7a), which is a surface moulded into a sequence of parallel ridges and grooves^59^. There were a few layers of GO nanosheets visible in the TEM image of GO in Fig. 7b. They have a lot of oxygen-containing functional groups on their surfaces and are flat because the van der Waals contacts between the GO layers were broken during sonification and sample preparation^60^. The surface analysis method helped to clarify the chemical composition (EDX). GO's EDX spectrum is displayed in Fig. 7c. In the prepared GO sample, the atomic percentages of carbon and oxygen are, respectively, 68.25 and 31.75. The produced membranes' surface morphology is examined using the scanning electron microscope (SEM) technique. SEM micrographs of the surface morphology of the plasma-pretreated PVA/PVP and various weight percentages (0.0, 0.1, 0.2, and 0.3 wt%) of GO-filled PVA/PVP polymeric nanocomposite membranes are shown in Fig. 7d–g. In the SEM image (Fig. 7d), the surface morphology of the PVA/PVP polymeric blend membrane's smoothness is clearly seen. The SEM image obtained for Fig. 7e depicts a homogenous distribution and fine dispersion of PVA/PVP:0.1 wt% GO. The white spots on the surface of membranes are spherical and increase in proportion to the amount of graphene oxide raised in the PVA/PVP composite. These bright spots are evenly distributed in a homogenous manner in the host PVA/PVP polymeric blend matrix with no agglomerations. Additionally, it was found that when graphene oxide (GO) was disseminated in the polymer matrix, small pieces formed since pure graphene has a low solubility in most solvents. When the filler concentration in the membrane is low, the filler nanoparticles are well dispersed from one another and distributed randomly and uniformly in the whole volume of the membrane. The dispersion is continued in the PVA/PVP:0.2 wt% GO and PVA/PVP:0.3 wt% GO composite membranes as shown in Fig. 7f,g, and the host polymer has not shown any signs of phase separation, which improves proton conductivity^61^.

**Figure 8:**
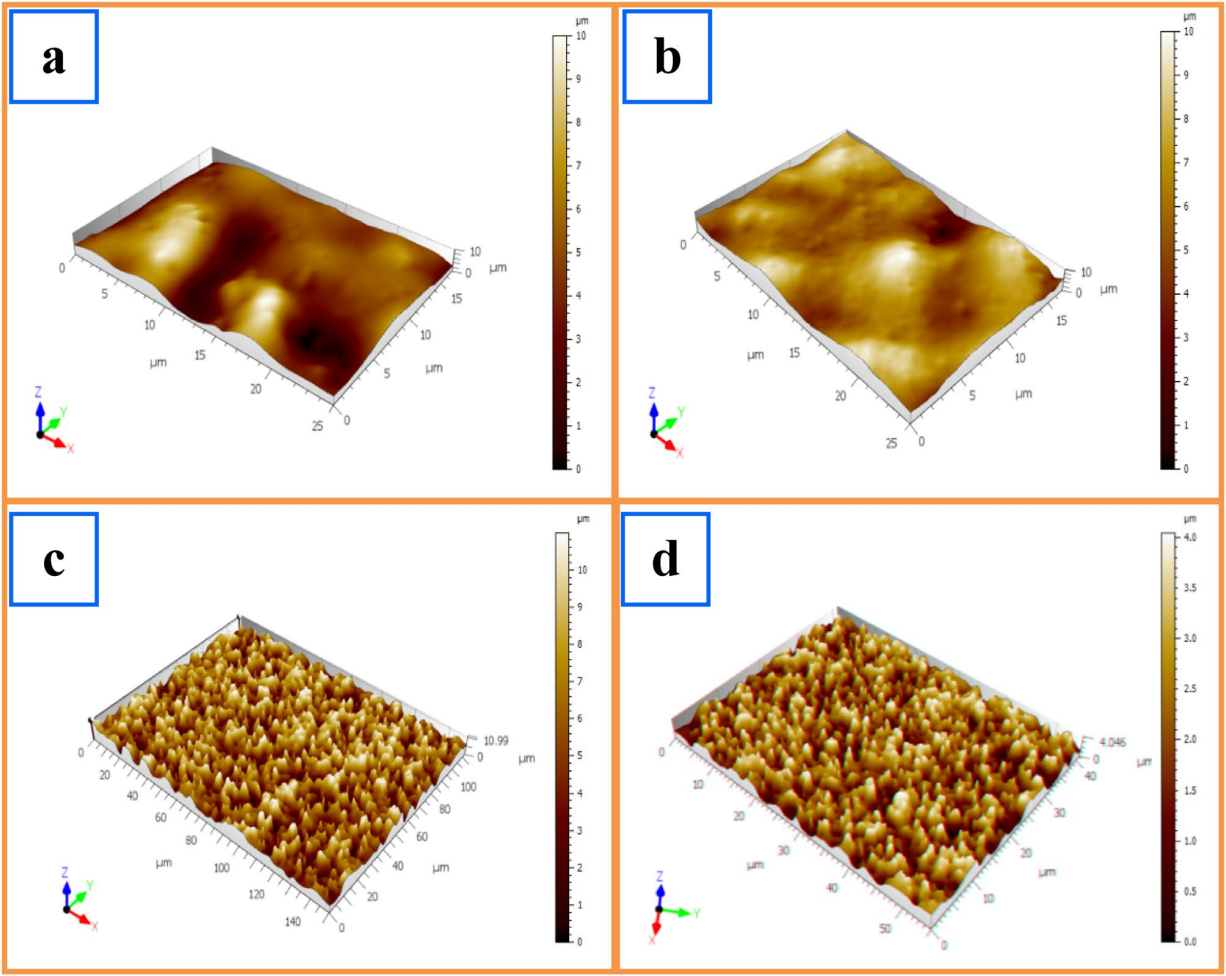
AFM study micrographs of (**a**) PVA/PVP blended polymers, (**b**) PVA/PVP: 0.1 wt% GO, (**c**) PVA/PVP: 0.2 wt% GO, and (**d**) PVA/PVP: 0.3 wt% GO composite.

Atomic force microscopy (AFM) study gives an idea of the surface modifications in a material. AFM was used to examine the effect of GO on the surface of the PVA/PVP blend. In Fig. 8, AFM studies are displayed; crystalline and amorphous portions of polymer surfaces can be distinguished from one another using the color changes in the micrographs. Dark parts could be associated with amorphous regions, whereas light spots could be related to higher crystalline regions. It is evident that the pure PVA/PVP surface, in comparison to PVA/PVP: xGO (x = 0.1, 0.2, and 0.3 wt%), nanocomposite membrane has a smoother surface, but the total roughness, as listed in Table 3, increases due to the increasing doping level of GO, which confirms the good miscibility of GO embedded in PVA/PVP. An increase in roughness can lead to an increase in surface area, which may result in higher AC conductivity due to increased contact between the membrane and the surrounding medium.

**Table 3:** Total roughness for pure and GO-doped membranes.

Sample	Total roughness R_t_ (nm)
PVA/PVP	30.15
PVA/PVP:0.1 wt% GO	45.63
PVA/PVP:0.2 wt% GO	69.90

### The structural properties of PVA/PVP-g-PSSA:xGO nanocomposite membranes

**Figure 9:**
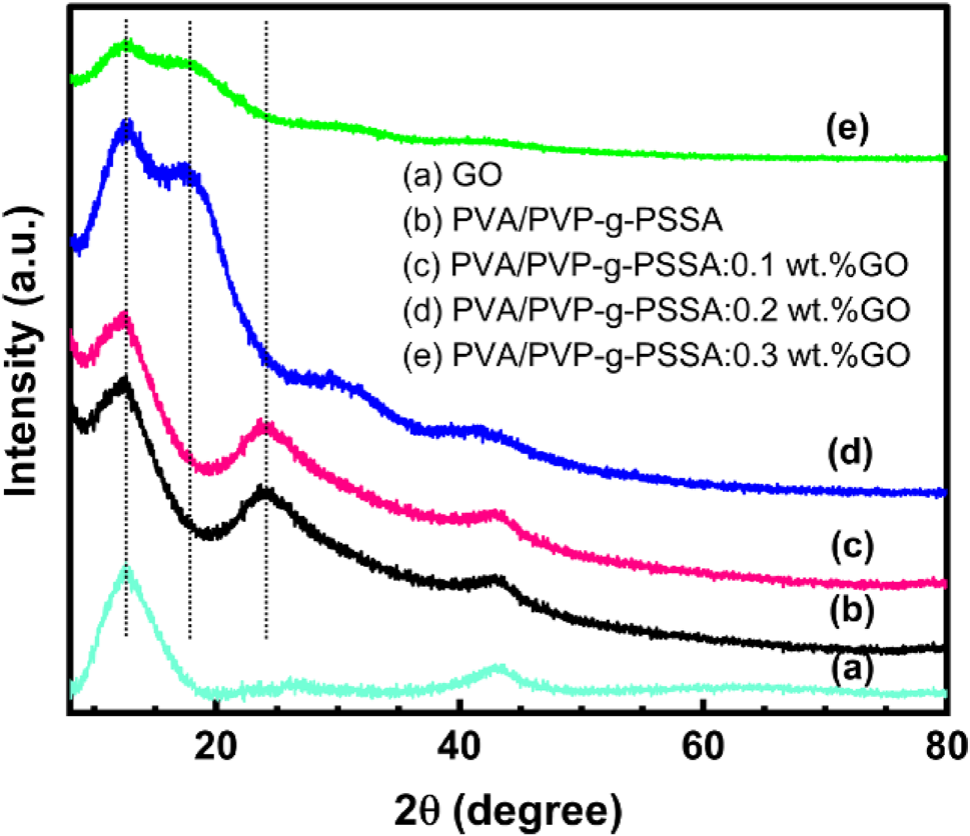
XRD pattern of (**a**) pure GO, (**b**) pure PVA/PVP-g-PSSA, and (**c**–**e**) 0.1–0.3 wt% GO: PVA/PVP-g-PSSA composite.

A study using X-ray diffraction (XRD) was done to determine how the structure of PVA/PVP-g-PSSA changed when nanoparticles of GO were added at various concentrations. Figure 9a–e shows the XRD pattern of pure GO, PVA/PVP-g-PSSA, and PVA/PVP-g-PSSA:x wt% GO (x = 0.1, 0.2, and 0.3) blend of polymeric nanocomposite membranes recorded from 5° to 70°. The XRD pattern for GO nanoparticles is shown in Fig. 9a; the principle peaks were observed at 2θ of 12.43° and 42.76°^62^ which are attributed to planes (001) and (100), respectively^63,64^. This observation confirmed the formation of GO. The XRD pattern of PVA/PVP-g-PSSA:x wt% GO polymeric nanocomposite membranes shows characteristic peaks at 13°, 23°, 30°, and 42°, indicating its semi-crystalline nature, which is in agreement with previous work^65–68^. As can be seen in the XRD pattern of PVA/PVP-g-PSSA: xGO membranes, there are no clear distinguishable peaks for GO. The absence of GO peaks in these XRD patterns is mostly due to the filler's (GO) good homogeneous dispersion in comparison to the host polymeric blend. When 0.3 wt% GO amounts were mixed into the PVA/PVP matrix, the second peak position (24°) shifted to a lower diffraction angle (18°), and a relative decrease in its intensity was detected. The decrease of the peak intensity of PVA/PVP with the increase of the GO ratio reveals a slight decrease in the degree of crystallinity as listed in Table 4, which was calculated from Eq. (8). The dispersion of GO with the blend polymers PVA/PVP was related to the decrease in crystallinity of the nanocomposite membranes with increasing GO content. Whereas GO diffuses in a PVA/PVP polymer mixture, the contact between PVA/PVP and GO lowers the intermolecular interaction between the polymer chains. The strength of the PVA/PVP diffraction peak lessens as the weight percentage of GO increases, implying that contact between PVA/PVP chains and GO particles reduces intermolecular interaction and increases the amorphous character of PVA/PVP. In amorphous regions, the chains are erratic and intertwined, whereas in crystalline sections, the chains are regularly ordered. As a consequence, molecular packing is weak in the amorphous state, and molecular chains can move more freely than in the crystalline form^69^. The crystal size D, and the d-spacing of the investigated composite membrane samples (PVA/PVP)-g-PSSA:x wt% GO were determined from Debye-Scherer Eq. (6) and Bragg's Law Eq. (7), respectively, and the results are listed in Table 4. The incorporation of GO nanoparticles into the PVA/PVP matrix resulted in the complete disappearance of the GO peak and the appearance of new amplified peaks, as shown in Fig. 9. These findings indicate uniform dispersion and good incorporation of GO nanoparticles in the PVA/PVP matrix, and the intensity of the peak became broader, indicating a decrease in the crystallization of this system.

**Table 4:** X-ray diffraction (XRD) data.

Sample	2 theta (degree)	FWHM (degree)	d-spacing (nm)	Crystallite size D (nm)	Degree of crystallinity (%)
GO	12.43	4.77	0.71	1.67	
PVA/PVP	12.66	4.83	0.70	1.73	45.54
PVA/PVP-g-PSSA:0.1 wt% GO	12.79	4.82	0.69	1.73	45.86
PVA/PVP-g-PSSA:0.2 wt% GO	12.43	6.81	0.71	1.23	36.97
PVA/PVP-g-PSSA:0.3 wt% GO	12.73	5.77	0.70	1.45	29.52

### Thermal stability (TGA)

**Figure 10:**
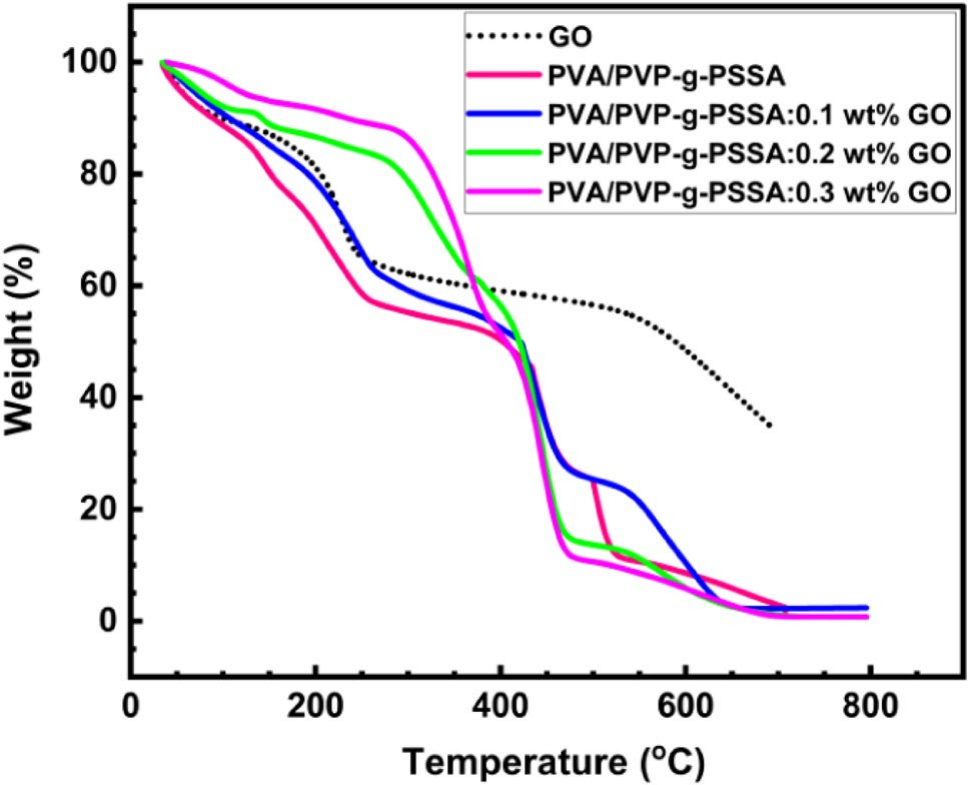
TGA thermograms of GO and PVP/PVA-g-PSSA:x wt% GO for various filler concentrations.

A thermogravimetric analysis was performed to assess the thermal stability of sulphonated copolymers. The weight loss curves for GO and all produced membranes are displayed in Fig. 10. Because of the elimination of physiosorbed water, GO demonstrates a progressive weight loss in the temperature range up to 100 °C, as shown in Fig. 10. The primary weight loss of GO was noted at a temperature range of 160–240 °C (40%)^70^. Some functional groups that contain oxygen are removed from GO, which results in weight loss that has been reported^71^. Water loss from the polymer matrix is essentially the first degrading phase for PVA/PVP membranes, and it is observed at a temperature range of 30–150 °C^72,73^. This stage is followed by a plateau that varies in temperature from 180 to 300 °C. At 250 °C, the weight loss percentage for the polymer blend was 45%, but for this blend that contained 0.3 wt% of GO, it was only 10% at that temperature. Desulphonation reactions were linked to this second step (180–300 °C)^74,75^. For the pure polymer blend, the third and primary stage of polymeric degradation occurred between 250 and 400 °C, reaching 300–400 °C for the PVA/PVP:GO composite samples. The primary polymeric chain and the most stable oxygen-containing groups, such as carboxyl groups, have been said to separate at this stage^76^. For every sample, the fourth stage of degradation took place between 420 and 480 °C. Eventually, it was shown that the degradation and carbonization of the polymer mixture were responsible for a peak that occurred between 500 and 650 °C^77,78^. PVA/PVP-g-PSSA membranes have chemical stability up to 180 °C with varying GO ratios, as seen by the TGA curve, making them appropriate for use in proton exchange membrane fuel cells.

### Water uptake (WU), swelling ratio (SR), and ion exchange capacity (IEC)

**Figure 11:**
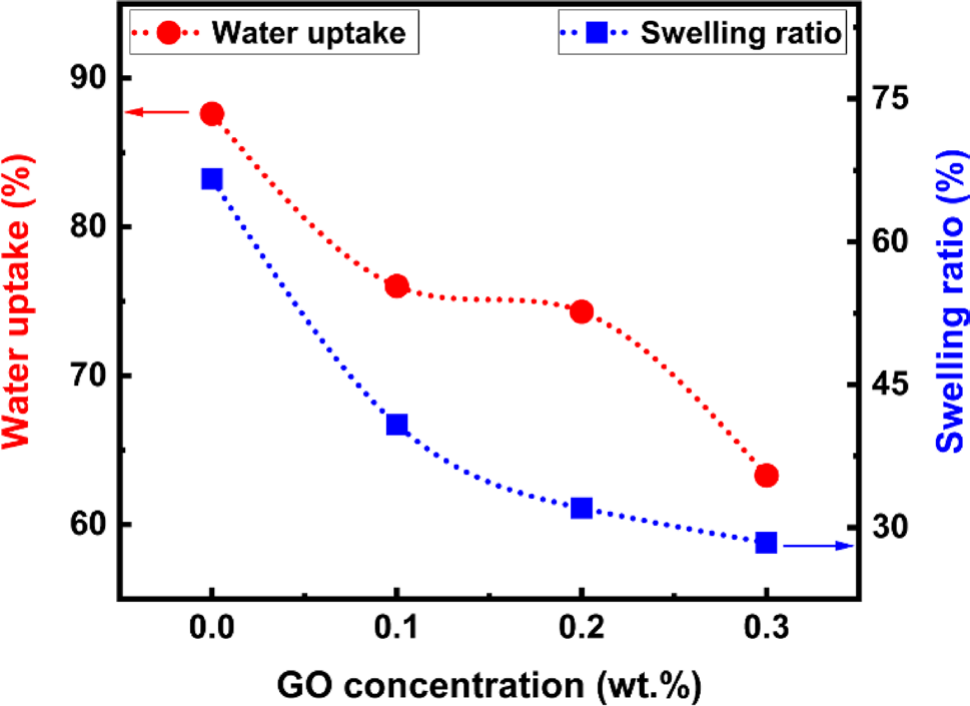
The variation of water uptake and swelling ratio with GO concentration.

In general, excessive water absorption promotes a loss of mechanical properties, although significant swelling from excess absorption subsequently dilutes the concentration of the OH-ionic group. However, the membrane becomes brittle when there is a lack of water. As a result, the conductivity and toughness values of the film are compromised. Equation 9 was used to compute the WU value, which was found to be 87% for a pure membrane. With the increase of GO content from 0.0 to 0.3 wt%, the WU decreased from 87 to 63%, as shown in Fig. 11. The WU of a composite membrane is affected by several factors, including the hydrophilicity of the used polymers and the porosity of the produced membrane. In the case of PVA/PVP-g-PSSA:x wt% GO composite membranes, the addition of GO can lead to a decrease in water uptake. This behavior can be explained by the chemical structures of PVA and PVP. PVA and PVP have high hydrophilicity because the hydrophilic characteristic of PVA shows that as the PVA percentage increases, so do the (–OH) groups, resulting in a high affinity with water molecules, giving nanofibers a higher moisture absorption capacity and a smaller contact angle. The carboxyl groups (–COOH) in GO interact with the hydroxyl groups in PVA when GO is introduced, and the quantity of hydroxyl groups reduces as the number of carboxyl groups increases^79^. The diminishing hydrophilicity of the membranes reflects this behavior. Furthermore, the SO_3_H groups block some water-absorbing sites, resulting in a decrease in the composite membranes' swelling ability^45^. Furthermore, the presence of GO can also lead to a decrease in porosity and an increase in tortuosity, which can further limit the diffusion of water molecules through the membrane. This is because GO can form a dense network within the polymer matrix, which can reduce the size and number of pores available for water uptake. Overall, the decrease in water uptake with increasing GO content in PVA/PVP-g-PSSA: wt% GO composite membranes can be attributed to a combination of factors, including the GO effect on the porosity and tortuosity of the membrane and the SO_3_H groups block some water-absorbing sites. The decrease in hydrophilicity of the membranes reflects this trend.

When the swelling was examined using Eq. (10) as shown in Fig. 11, the pure PVA/PVP-g-PSSA sample showed a swelling ratio of 66.6% without any GO additions. However, the samples including GO showed a swelling ratio of 28.4% for PVA/PVP-g-PSSA: 0.3 wt% GO. From these results, it can be concluded that the addition of GO to PVA/PVP-g-PSSA reduces the swelling ratio; this is more likely owing to the GO's crosslinking effect. The COOH and OH groups on GO can also create hydrogen bonds with PVA and PVP macromolecules, in this situation, the GO serves as physical crosslinking points, limiting the swelling of the composite membranes and resulting in a decreased swelling ratio^80^. In general, plasma grafting can reduce the water uptake and swelling ratio of a material. The grafted polymer layer acts as a barrier, preventing direct contact between the substrate and water molecules. This reduces the diffusion of water into the material, leading to lower water uptake and swelling. The plasma exhibited polymer deposition, with the top layer resembling a hydrophobic surface with low water sorption. However, because this layer was so thin, water was easily absorbed through the opposite side of the membrane, resulting in a minor decrease in water content from 87.6 to 63.3%^81^.

IEC is an essential characteristic that influences many properties of PEMs and provides information on the number of functional groups (SO_3_) in the membrane, which may be thought of as a method of getting the membrane's proton conductivity. Table 5 displays the IEC values of all prepared membranes. As revealed from the table, the IEC has values of 1.12 and 1.9 meq/g for PVA/PVP-g-PSSA and PVA/PVP-g-PSSA: 0.3 wt% GO, respectively. The increase in GO concentration increases the degree of grafting (as mentioned earlier), which in turn leads to an increase in the available sites to graft SO_3_ groups in the polymer chains. The amount of SO_3_ groups in the prepared samples generally increases as the DOG increases. At increasing styrene concentrations, more benzene rings come into contact with sulfonic acid groups, resulting in more SO_3_ groups in the membrane. In addition, by increasing the IEC, the proton conductivity also increases.

**Table 5:** Water uptake, swelling ratio, and IEC values of all prepared membranes.

Sample	Water uptake (%)	Swelling ratio (%)	IEC (meq/g)
PVA/PVP-g-PSSA	87.6	66.6	1.12
PVA/PVP-g-PSSA:0.1 wt% GO	76.0	40.8	1.14
PVA/PVP-g-PSSA:0.2 wt% GO	74.3	32.1	1.78
PVA/PVP-g-PSSA:0.3 wt% GO	63.3	28.4	1.91

### Proton conductivity

**Figure 12:**
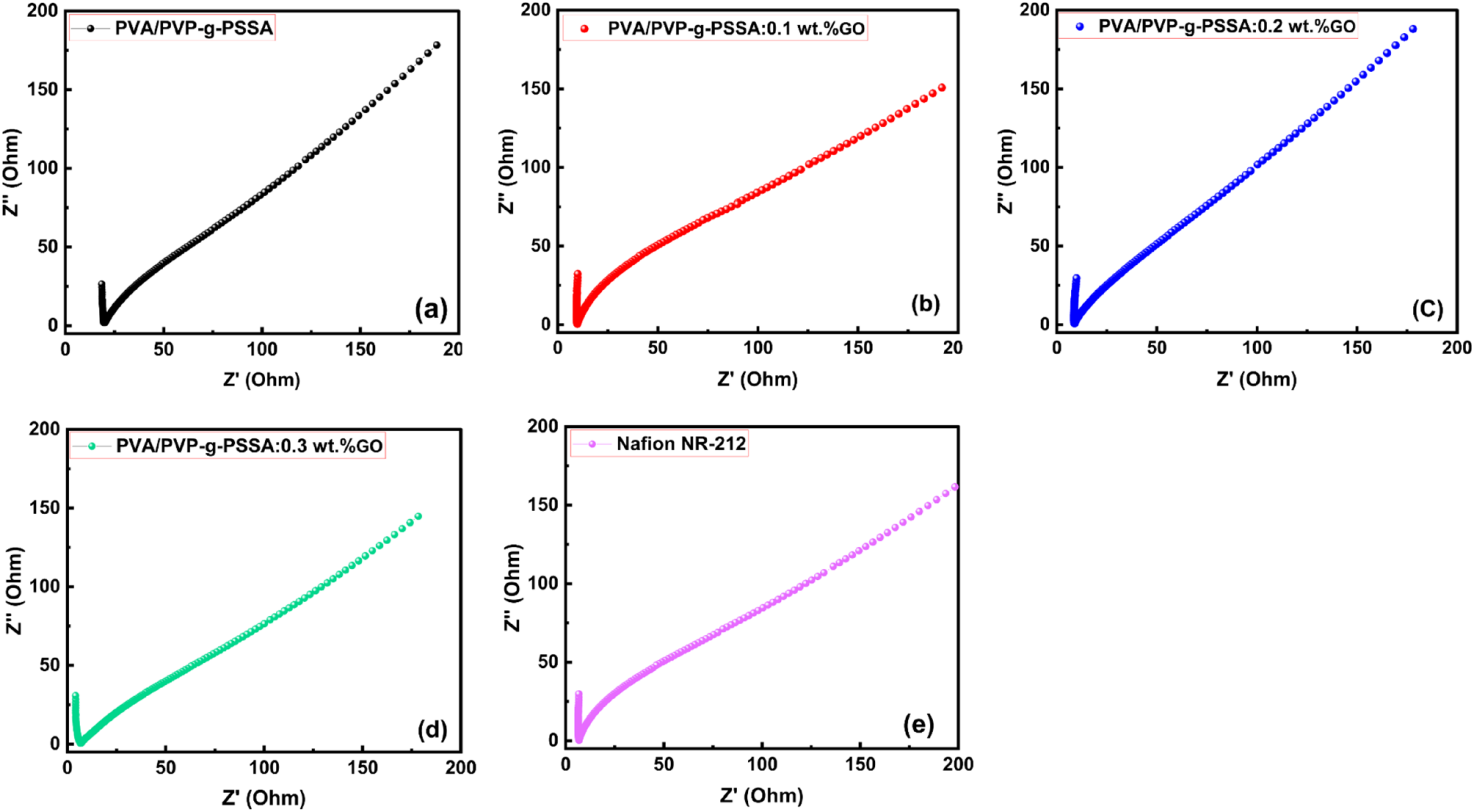
Cole–Cole plot of prepared membranes (**a**) PVA/PVP-g-PSSA, (**b**) PVA/PVP-g-PSSA:0.1 wt% GO, (**c**) PVA/PVP-g-PSSA:0.2 wt% GO, (**d**) PVA/PVP-g-PSSA:0.3 wt% GO, and (**e**) Nafion NR-212.

Figure 12a–d shows the impedance spectra of grafted PVA/PVP-g-PSSA pure and PVA/PVP-g-PSSA:x wt% GO membranes, while Fig. 12e shows the impedance spectrum of the Nafion NR-212 membrane for comparison. All of the samples were completely bloated with water. This figure clearly shows that, for PVA/PVP-g-PSSA membranes, the resistance to conducting ions reduces as GO content increases. For PVA/PVP-g-PSSA:x wt% GO (x = 0.1, 0.2, and 0.3) with IEC of 1.14, 1.78, and 1.91 meq/g, respectively. The impedance spectra showed that the resistance decreases with increasing content of GO from 0 to 0.3 wt%, and in turn the value of conductivity increases. The resistance of the prepared membranes derived from impedance spectra and can be then transformed into proton conductivity using the following equation:

15 $$\sigma =\frac{L}{RA}.$$

where $\sigma$ is the DC conductivity, *L* is the thickness of the membrane, *R* is the measured resistance, and *A* is the effective area of the blocking electrode.

**Figure 13:**
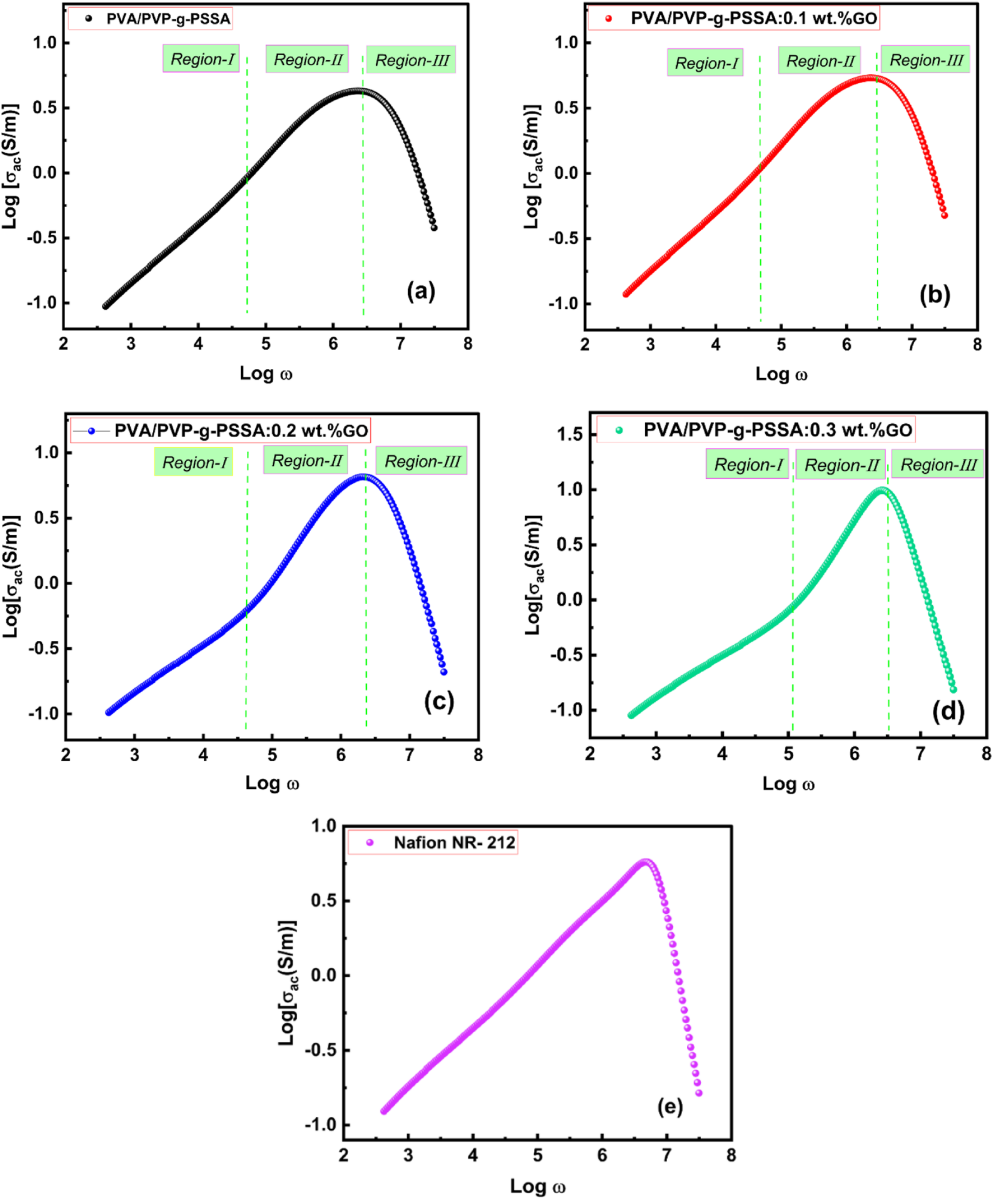
AC conductivity versus frequency at room temperature of prepared membranes (**a**) PVA/PVP-g-PSSA, (**b**) PVA/PVP-g-PSSA:0.1 wt% GO, (**c**) PVA/PVP-g-PSSA:0.2 wt% GO, (**d**) PVA/PVP-g-PSSA:0.3 wt% GO, and (**e**) Nafion NR-212.

It is well known that the σ of the membranes and the water absorption affect the IEC values. In order to reduce the interfacial energy, the attachment of water to the acid functionality in the membrane causes a modified cluster structure that transforms into spherical water pools. Water can pass through the membrane's water channel and allow protons to travel through as well. For all samples, the AC conductivity (Log σac) is shown in Fig. 13a–d as a function of frequency (Log ω). As shown in Fig. 13, three zones were observed for all samples examined. Because of a drop in polarization, the AC conductivity values rose linearly with frequency in both the I and II zones, with varying slopes^82^. The AC conductivity was represented by Jonscher's universal power law as,

16 $${\sigma }_{ac}\left(\omega \right)=A{\omega }^{s}.$$

where ω is the angular frequency, s is the frequency exponent, and A is a temperature-dependent constant identifying the polarizability strength (0 < *s* < 1). As shown in Fig. 13, according to the Jonscher's power law, the obtained curves were fitted at the low frequency zones (I & II) and *s* < 1 revealing that hopping is the conducting mechanism in the I and II zones^83,84^. The conductivity decreased as the frequency was increased further (region III). This performance is connected to the applied field, which interferes with the hopping mechanism, which causes the conductivity to drop as the frequency is increased^85^. The proton conductivity values at room temperature were listed in Table 6. The table shows that a higher GO doping percentage increases the proton conductivity of PEMs. This is due to the well-dispersed GO, which allows the PVA/PVP-g-PSSA membranes to have a high surface area and a significant majority of oxygen-containing groups. Doping PVA/PVP-g-PSSA with GO nanoparticles, on the other hand, enhances the microstructure and provides more proton exchange channels^86^.

**Table 6:** Methanol permeability, proton conductivity, and efficiency measured values for all prepared samples.

Sample	Methanol permeability (cm^2^/s)	Proton conductivity (mS/cm)	Efficiency (S cm^−3^ s)
PVA/PVP-g-PSSA	1.82 × 10^−6^	42.78	2.35 × 10^4^
PVA/PVP-g-PSSA:0.1 wt% GO	1.21 × 10^−6^	53.84	4.45 × 10^4^
PVA/PVP-g-PSSA:0.2 wt% GO	1.11 × 10^−7^	65.52	5.90 × 10^5^
PVA/PVP-g-PSSA:0.3 wt% GO	1.03 × 10^−7^	98.90	9.60 × 10^5^
Nafion NR-212	1.63 × 10^−6^	57.72	3.54 × 10^4^

### Frequency and GO content effects on the dielectric constant

**Figure 14:**
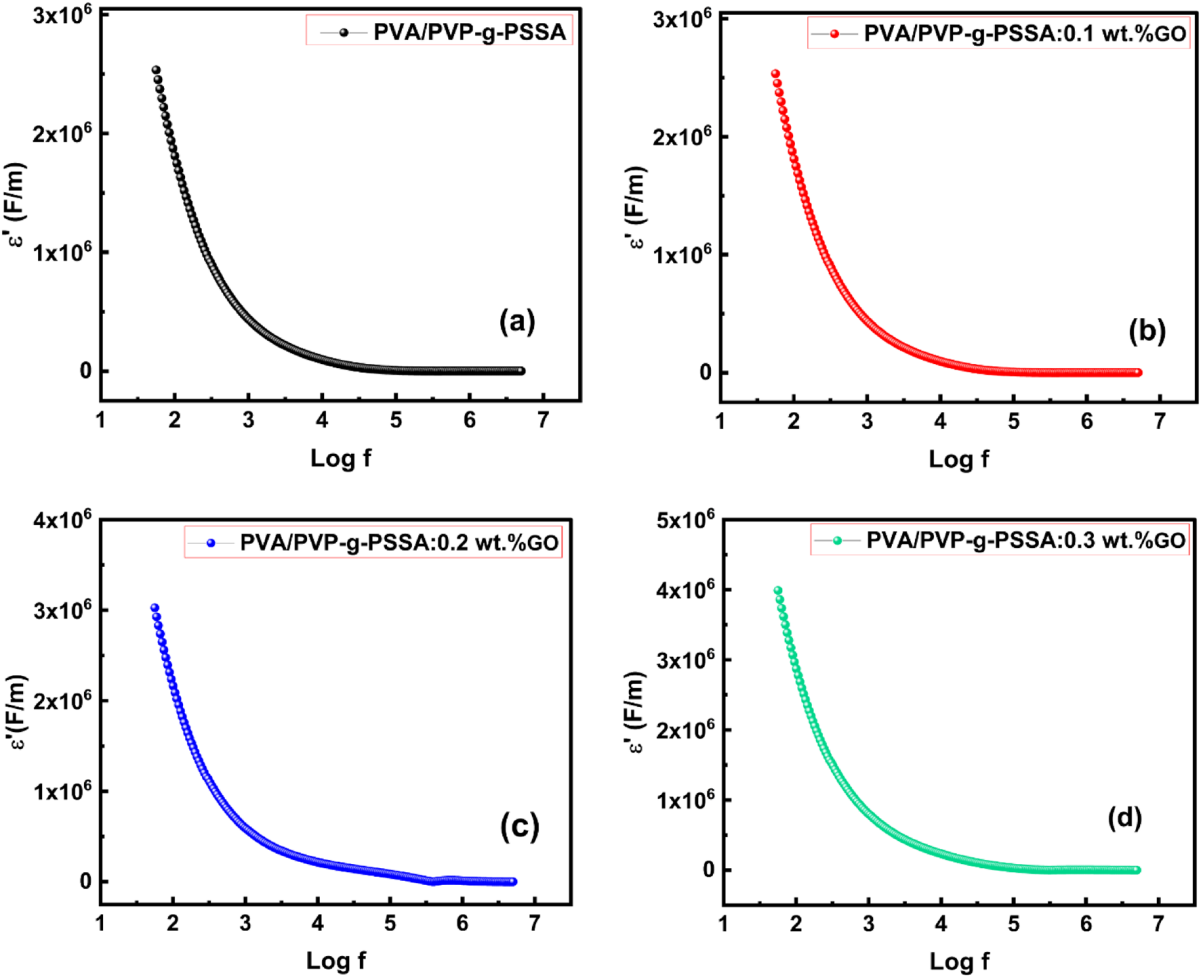
The frequency dependence of ε' for PVA/PVP pure and doped with different GO concentrations (0.1, 0.2, and 0.3 wt%) (**a**–**d**, respectively) at room temperature.

**Figure 15:**
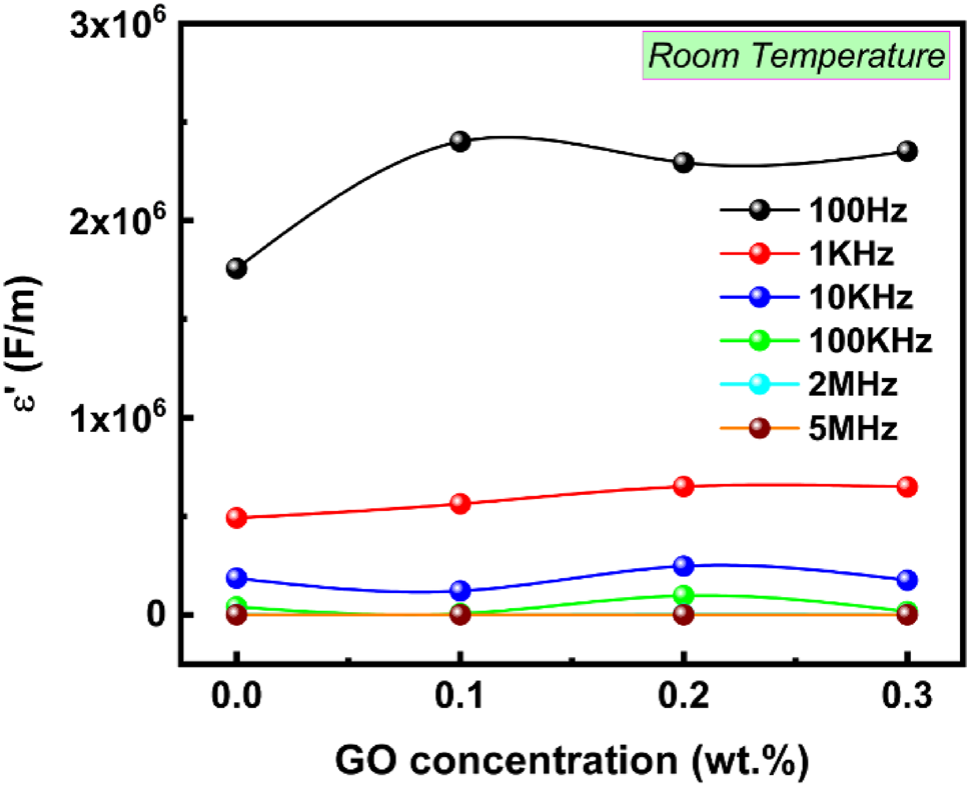
Dielectric constant εʹ vs. GO content at different frequencies.

Figure 14 investigates the dielectric constant which reveals important details about the composition, the grain and grain boundary, and the transport characteristics of the compounds. The dielectric constant can also be used to gauge a material's capacity to store electrical energy. An irregular frequency-dependent decrease in the dielectric constant was observed. Maxwell–Wagner interfacial polarization model^87^ describes how this behavior occurs. Because the dipoles have adequate time for interfacial polarization, it is evident that low frequencies have a stronger influence on $\varepsilon'$ than high frequencies. Although this is easily observable in the high-frequency range, dipoles do not have enough time to follow the fast change of the external field at high frequencies. For GO concentrations up to 0.2 wt% GO, a significant rise in the dielectric constant was seen for all frequencies. Following this, the dielectric constant indicated a little rise in GO concentration. This might be connected to several polarization mechanisms, each of which contributes differently to the polarization and is influenced by the applied electric field. Generally, this increase in εʹ with GO concentration, as observed in Fig. 15, showed an enhancement in the tested sample's capacity to store electric energy.

### Frequency and GO ratio effects on dielectric loss

**Figure 16:**
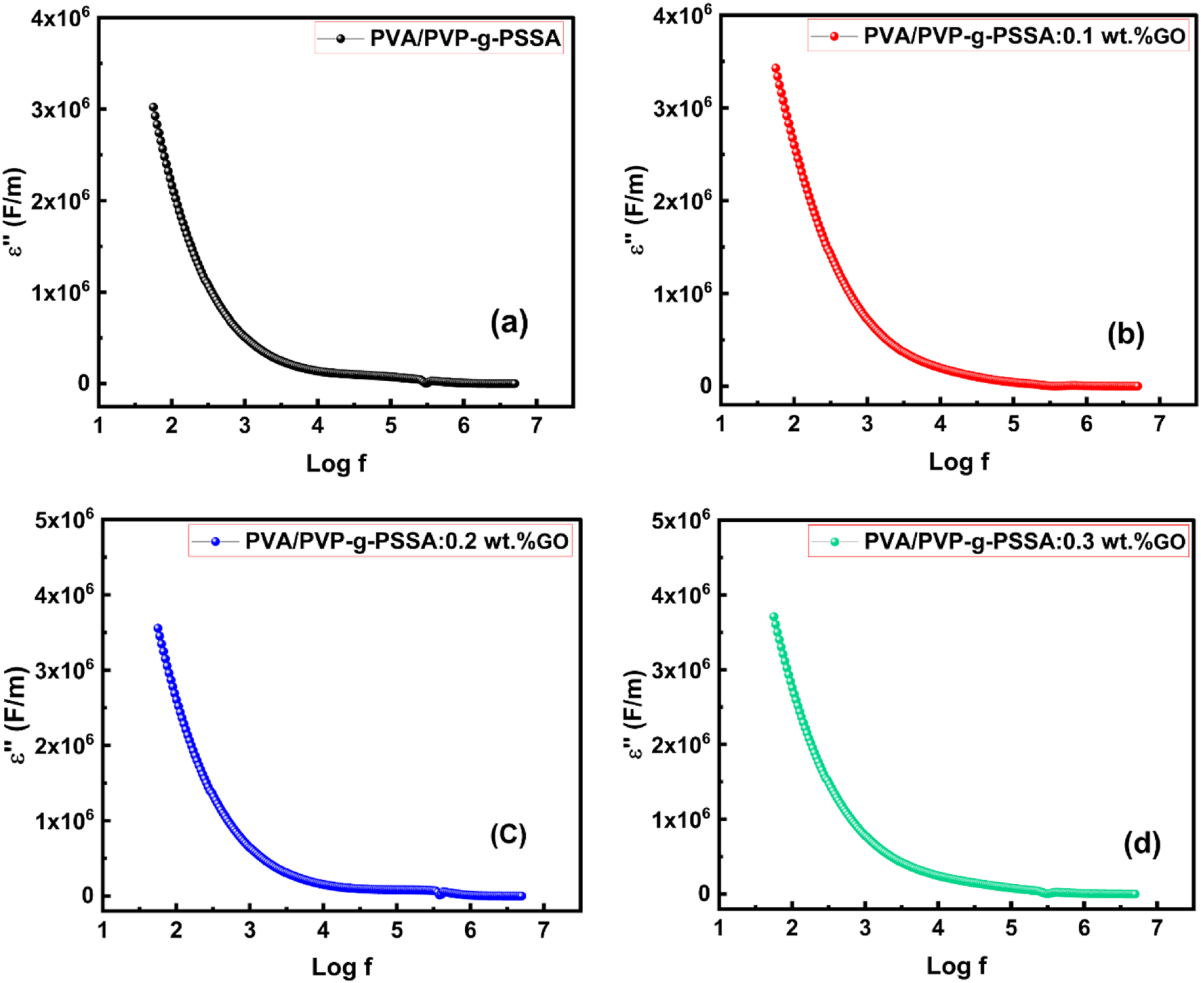
The frequency dependence of εʺ for PVA/PVP-g-PSSA pure and doped with different GO concentration (0.1, 0.2 & 0.3 wt%) at room temperature.

**Figure 17:**
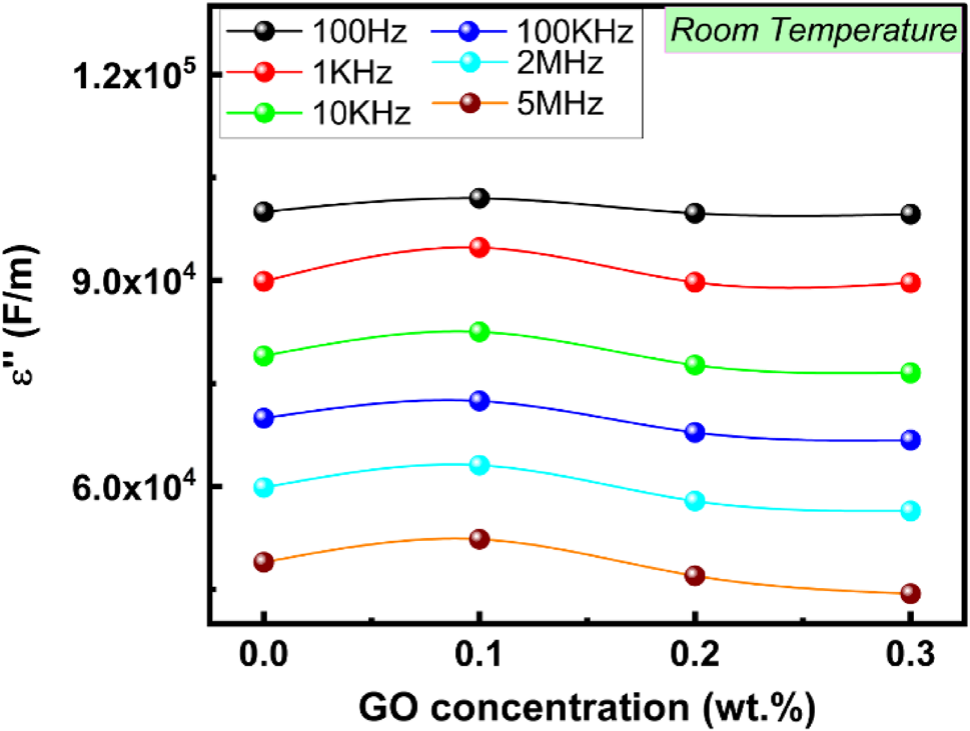
Dielectric loss εʺ vs. GO concentrations at different frequencies.

Figure 16 depicts the relationship between the frequency for PVA/PVP-g-PSSA:GO examined samples and the dielectric loss (εʺ). In general, ion mobility, dipole relaxation, and interfacial polarization are the three factors that cause dielectric loss in polymers. The dielectric loss consistently displays the same trend for each sample examined. At low frequencies, the dielectric loss begins to rapidly decrease, then εʺ gradually decreases in the frequency range (1–100 kHz) and after that remains constant with frequency in the high-frequency region. There was a slight drop in εʺ in the high-frequency range as compared to the lower frequencies due to the polarization's various characteristics^88^. The fluctuation of εʺ with GO concentration at various frequencies is shown in Fig. 17. Both εʺ and εʹ with GO concentration exhibited a similar pattern of action. From the figure, it can be seen that εʺ has slightly increased, when GO reaches 0.1 wt%. Following that, it seemed stable at lower frequencies with GO concentrations and declined at higher frequencies with higher GO concentrations. This demonstrates that for GO concentrations greater than 0.2 wt%, the influence of interfacial polarization is less substantial at high frequencies and becomes more consistent at lower frequencies.

### Methanol permeability

Methanol permeability is the resistance of a membrane to methanol passing through it between a cathode and an anode. The passage of methanol, hydrogen, or oxygen gas through the PEM degrades the cell's performance and degrades the PEM. In general, the fuel permeability through PEM is believed to be correlated to the structure of the membrane. Equation (12) was used to calculate the produced membranes' methanol permeability and the values listed in Table 6. As seen in Table 6, it is evident that methanol permeability declines as GO concentration increases. The methanol permeability for as-prepared samples decreases from 1.82 × 10^−6^ to 1.03 × 10^−7^ cm^2^/s with increasing GO content. While the lowest value was recorded for the sample PVA/PVP-g-PSSA: 0.3 wt% GO (1.03 × 10^−7^ cm^2^/s), which is less than the Nafion NR-212 permeability (1.63 × 10^−6^ cm^2^/s), in accordance with Shaari et al.^89^. In light of the membrane microstructure between GO and PVA/PVP-g-PSSA, the low methanol permeability can be explained; the added GO filler particles significantly obstruct the connected hydrophilic passageways in the PVA/PVP-g-PSSA composite membrane. Such interfacial interaction between the GO and PVA/PVP-g-PSSA: wt% GO composite membrane reduces the methanol permeability. The blocking effect of the GO filler lowers the methanol permeability by preventing methanol from migrating through the membrane. Methanol penetration in the membrane has a direct impact on PEMFC efficiency because catalyst poisoning can occur due to the methanol crossed-over leading to a reduction in the electrocatalytic activity of cathode catalysts^90^. As a result, PEM benefits from decreased methanol permeability.

### Membrane efficiency

The efficiency factor, defined as the ratio of proton conductivity to methanol permeability, was used to assess membrane performance in terms of both proton conductivity and methanol permeability^91^. The efficiency factor is widely cited as a measure of membrane applicability by comparing its value to that of commercial materials. A greater efficiency value indicates improved application in DMFCs. The efficiency of PVA/PVP-g-PSSA, PVA/PVP-g-PSSA:x wt% GO membranes and Nafion NR-212 is shown in Table 6. The presence of GO greatly enhanced the efficiency of the PVA/PVP-g-PSSA membranes. These findings were ascribed to the inclusion of GO, which influenced the lowering of methanol crossover. The maximum efficiency was observed at 0.3 wt% GO loading. The 0.3 wt% GO membrane has an efficiency rating that is roughly four times that of Nafion NR-212. As a result, PVA/PVP: GO membranes are unquestionably attractive materials for DMFC applications.

## Conclusions

A new proton-conducting polymer electrolyte membrane based on a PVA/PVP blend (1:1) mixed with different ratios of graphene oxide (GO) was successfully made under the influence of Cold Atmospheric Plasma (CAP). The SEM results showed a higher dispersion of membranes formed by nanocomposite, in contrast to the AFM data, which showed an increase in overall roughness with increasing the proportion of GO. The FTIR study verified the chemical complexity of the polymer membranes, the grafting process, and the sulfonation process. The obtained samples' semicrystalline nature was shown by XRD, and by gradually raising the GO concentration, the degree of crystallinity was steadily reduced. From the TGA results, PVA/PVP-g-PSSA membranes with varied GO ratios are chemically stable up to 180 °C, making them appropriate for proton exchange membrane fuel cells. Proton conductivity has increased as GO concentration has increased, with PVA/PVP-g-PSSA: 0.3 wt% GO nanocomposite membrane having the greatest value (98.9 mS/cm). The addition of GO also improved the methanol permeability of the membranes, where the lowest permeability value is 1.03 × 10^−7^ cm^2^/s for PVA/PVP-g-PSSA: 0.3 wt% GO nanocomposite membrane, which is inferior to that of Nafion NR-212. The study found that these PEMs outperform Nafion NR-212 in several ways, including lower methanol permeability and higher proton conductivity. Finally, the novel polyelectrolytic membranes based on PVA/PVP-g-PSSA: 0.3 wt% GO have great potential for future low-cost DMFC applications.

## Data Availability

The datasets used and/or analysed during the current study are available from the corresponding author on reasonable request.
